# An empowerment-based, healthy dietary behavioral intervention to ameliorate functional constipation

**DOI:** 10.3389/fnut.2023.1043031

**Published:** 2023-03-27

**Authors:** Xuesong Wang, Xiaohui Zhong, Dongsong Liu, Hong Cao, Jing Chen, Qinyue Wang, Yanping Xia, Feng Zhang

**Affiliations:** ^1^Department of Orthopedics, Affiliated Hospital of Jiangnan University, Wuxi, Jiangsu, China; ^2^Wuxi School of Medicine, Jiangnan University, Wuxi, Jiangsu, China; ^3^Department of Endocrinology, Affiliated Hospital of Jiangnan University, Wuxi, Jiangsu, China; ^4^Department of Nutrition, Affiliated Hospital of Jiangnan University, Wuxi, Jiangsu, China

**Keywords:** empowerment, behavior, constipation, intervention, diet

## Abstract

**Objective:**

To explore the boost effect on ameliorating functional constipation in elderly patients through empowerment-based, healthy dietary behavioral intervention.

**Design:**

In this randomized parallel group study, elderly patients with functional constipation were recruited and assigned to the experimental and control groups at a ratio of 1:1. The control group received routine intervention. The experimental group received 3-month empowerment-based intervention. The results were evaluated based on the Healthy Lifestyle and Personal Control Questionnaire (HLPCQ) and Cleveland Clinic Constipation Score (CCS). GraphPad Prism (Version 9) software was used for the statistical analysis.

**Setting:**

As the world's population ages, functional constipation in the elderly has attracted widespread attention. The practical behavioral intervention to ameliorate constipation are worth exploring.

**Participants:**

Sixty elderly patients with functional constipation.

**Results:**

The study results showed no significant difference in the baseline data between the two groups (*P* > 0.05). After the intervention, the scores of HLPCQ (77.90 ± 14.57 vs. 61.11 ± 13.64) and CCS (7.48 ± 3.73 vs. 9.70 ± 3.07) in the experimental group were significantly higher than those in the control group (*P* < 0.05).

**Conclusion:**

The results showed that empowerment-based intervention can effectively strengthen the healthy dietary behavior of elderly patients. Through patient empowerment, the subjective initiative and willingness to communicate were boosted in the experimental group. Their symptoms of functional constipation improved considerably better than in the control group.

## Introduction

Constipation, as one of the geriatric syndromes, affects up to 11.7% of older citizens every year, and its prevalence is still rising (1). The global prevalence of functional constipation without organic lesions is about 8.7–11.6% when Rome IV criteria were used (2). Complications of functional constipation, such as fecal impaction, hemorrhoids and anal fissures, can easily lead to impaired ability to work. About 70% of patients complain that constipation will bring trouble to their daily life and social relations (3). Regardless, no exact cause of functional constipation, which may be the result of multiple factors, has been identified. In general, the potential risk factors associated with functional constipation are not only demographic factors (mainly higher age, women and low education) but also lifestyle factors and behaviors (mainly lacking fiber and liquids) (4). For example, survey results showed that patients have the intention of eating healthily, but they lack a certain level of cognition about dietary choices (5).

Therefore, a concept of empowerment is introduced here. In the medical field, empowerment means that people have the ability to control their own lives and improve their health by enhancing their ability to solve important problems (6). Responding to the challenge of chronic disease, the Chinese government has integrated patient empowerment into chronic disease management programs (7). Intervention based on empowerment theory has been widely used in the management of chronic diseases, such as hypertension (8), chronic kidney disease (9) and diabetes mellitus (10). Nevertheless, the existing health education and empowerment methods for the elderly patients are obscure and boring, and difficult to adhere to for a long time. Dietary intervention is usually listed as the first-line or the first step of treatment for constipation (11), which is also consistent with the Chinese preference for dietary therapy (12). In addition, a high dietary fiber diet has long been recommended for the improvement of constipation (13). Accordingly, this study aimed to empower patients to adopt healthy dietary behaviors by combining the educated demonstration of high-fiber food models and cards, and ultimately achieved the outcome of amelioration of constipation.

## Methods

### Study design

**Figure 1:**
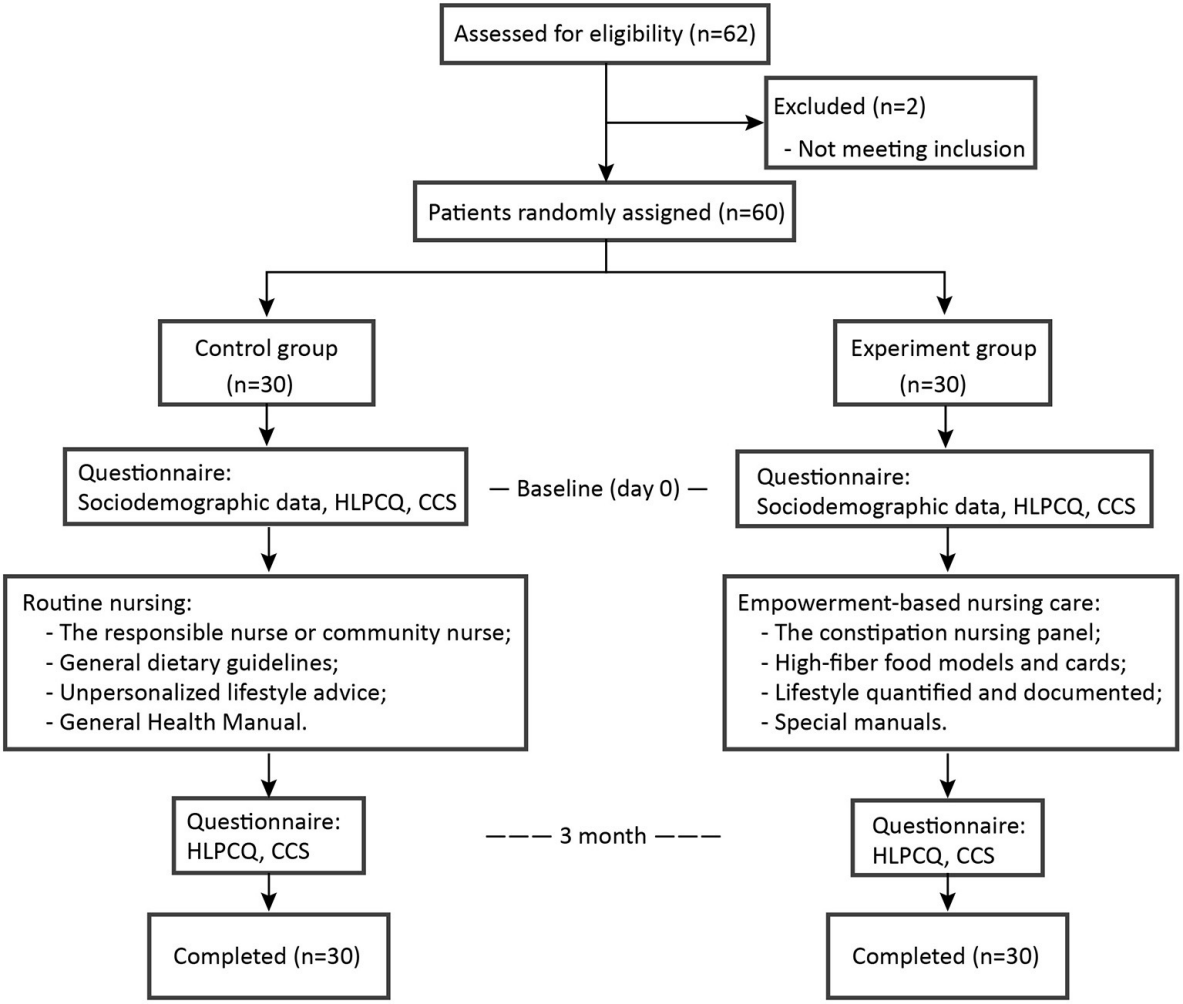
Flowchart of the study.

This randomized parallel group study was conducted at a tertiary hospital in Wuxi, China from 2020 to 2021. The study was single-blinded for the participants but not for the researchers, and a randomized list was generated by a statistician who used a computer to determine the group assignment of every participant. The main outcome indicator was healthy behavior, and the secondary outcome indicators were constipation symptoms. See Figure 1 for the flow chart of this study.

### Participants

Sixty participants with functional constipation were recruited from July to October 2020. A total of 30 participants were allocated to the experimental group, which received empowerment-based behavioral intervention for 3 months and 30 participants in the control group were given routine care.

The inclusion criteria were as follows: (1) older adults (more than 65 years of age), (2) the Rome IV criteria for functional constipation including dyssynergic defecation and slow colonic transit, officially published by the American Gastroenterology Association in 2016 (14), (3) availability for telephone follow-up; (4) capability to give written informed consent.

The exclusion criteria were patients (1) with evolving cancer, serious cognitive problems, a psychiatric disease, or a serious medical or health condition that would hinder their ability for defecation, (2) having received abdominal and intestinal surgery, (3) with intestinal obstruction by electronic enteroscopy, (4) with rectal prolapse and internal hemorrhoids rated as grade 3–4 and (5) taking drugs affecting the intestinal motility.

### Sample size

The healthy behavioral score of HLPCQ was used to estimate sample size, which was calculated on PASS software (version 15) and based on the following formula: *n*_1_ = *n*_2_ = [(Z_1_ – Z_α/2_ + Z_1−β_)^2^^*^ ($\sigma_{1}^{2}$ + $\sigma_{2}^{2}$)]/δ^2^. In the formula, n_1_ and n_2_ are the sample content required by the two groups of samples. In the pilot experiment, the HLPCQ score of the experimental group and control group after intervention were 66.15 ± 13.84 and 46.58 ± 6.36, respectively. The standard deviation σ_1_ of conventional measures to improve HLPCQ score was about 6, and the standard deviation σ_2_ of empowerment intervention to improve HLPCQ score was estimated to be 14. If the HLPCQ score was 10 points higher than that of control group after intervention, the intervention was considered to be popularized. Thus, δ = 10. With α = 0.05, β = 0.10, Power = 90%, two-sided test, and checking the statistical table, values of Z_1_-_α/2_ = 1.96 and Z_1−β_ = 1.28 were obtained. Therefore, the required number of samples was 26 cases. Considering that the attrition rate was 10%, the actual number of subjects recruited into the groups was 30 cases.

### Interventions description

The intervention was mainly based on empowerment theory and was conducted around three elements of empowerment. Patients in the experimental group diagnosed with functional constipation were managed by a constipation treatment panel, which consisted of 6 nurses, 4 doctors and 3 nutritionists who had qualification certificates and rich work experience. In addition, 4 graduate students provided assistance. The research contents to meet the three elements of empowerment were as follows:

(1) Internal factors: improve patients' internal beliefs and attitudes

Patients are encouraged to express their feelings and dietary preferences. Through explanation and goal setting, patients can obtain a clear understanding of functional constipation and establish confidence in overcoming constipation.

(2) Interaction elements: enhances the interaction between patients and the environment and promotes the acquisition of patients' knowledge, skills and resources

Medical staffs and patients worked together to analyse the causes and mainly sorted out the following factors of functional constipation: (1) Medical staff: lack of cooperation of specialized nurses, doctors and nutritionists; (2) patient: the general diet regulation failed, the large adjustment cannot be adhered to and the healthy dietary behavior was poor; (3) methods: lack of individualized home assessment and professional guidance; (4) resources: lack of health education materials for constipation; lack of resources and skills to effectively ameliorate constipation.

The corresponding intervention to promote patient empowerment were as follows: (1) medical staff: establishment of a constipation treatment panel; (2) patient: establishment of a plan based on empowerment theory, interaction with patients by high-fiber food models and cards during health education and testing of patients' knowledge and familiarity diet matching skills; (3) methods: the patients were required to record their 24 h fluid intake, dietary intake, defecation and exercise contents in the healthy dietary behavior log. In addition, health lectures were carried out and a WeChat official account pushed relevant knowledge on the phone; (4) resources: provided patients with paper and electronic education materials and prebiotic food.

(3) Behavior elements: promote patients to adopt healthy dietary behavior

The nutritionist designed the patient's recipe based on the recommended nutrient intake in the Table 1. Health dietary behaviors were logged by patients at home as shown in Table 1 and the medical staff evaluated whether the patient adhered to the health dietary behavior. The total dietary fiber intake recommended by the US Food and drug administration is 20–35 g/day, in which the insoluble and soluble dietary fibers account for 70–75% and 25–30%, respectively (15). On this basis, the doctors provided diet matching demonstration and functional food selection suggestions based on the degree and characteristics of patients' constipation. For example, for patients with normal body mass index (BMI) and without hyperglycaemia, the proportion of insoluble fiber diet from roots and grains must be increased. Patients with high BMI or hyperglycaemia are suggested to increase their intake of soluble fiber diet from apple, kelp and flaxseed. The slightly sweet xylooligosaccharides are given to patients who cannot accept meal replacement powder and fruit and vegetable diets. In this study, xylooligosaccharides were provided to all participants free of charge. The patients followed the recipe and completed a 24-h dietary recall questionnaire. According to the weekly repeated evaluation, the patients were reminded once their diet was not met the requirement of the recommended nutrient intake. In addition, abdominal massage and exercise guidance were the same as those in the control group. In general, the physician's intervention was individualized for each patient and the same treatment was given to each of the subjects included in the study.

**Table 1:** Health dietary behavior log.

**Item**	**Time**	**Details**
**Meals**		
Breakfast	e.g., 8:00	e.g., A box of milk 150 g + an egg 50 g + sweet potato 200 g + vegetable salad 100 g or protein 200 g + high-quality carbon water 200 g + dietary fiber 100 g
Lunch	e.g., 12:00	e.g., Dietary fiber 200 g + high-quality carbon water 150 g + protein 200 g
Dinner	e.g., 18:00	e.g., High-quality carbon water 100 g + vitamin 150 g + dietary fiber 200 g
Extra meal	e.g., 21:00	e.g., An apple

### Evaluation tools

The Healthy Lifestyle and Personal Control Questionnaire (HLPCQ) was developed by Darviri in 2014, and it focuses on the evaluation of the healthy behavior of patients (16). The total score of HLPCQ was 104 points, with higher scores indicating better behavior of patients in managing their symptoms. It has been reported that the Chronbach's α coefficient of HLPCQ was 0.827, and the result of Bartlett's test of Sphericity was *P* < 0.001, and the KMO value was 0.797 by Kaiser-Meyer-Olkin measurement, suggesting a good reliability and validity in applying HLPCQ (16). In addition, the Cleveland Clinic Constipation Score (CCS) was designed by the American Gastroenterology Society, and it is used to assess patients' gastrointestinal and anorectal symptoms (17). The total score of CCS was 30 points, with higher scores indicating more severe constipation of patients. The Chronbach's α coefficient of CCS was 0.839, and the result of Bartlett's test of Sphericity was *P* < 0.001, and the KMO value was 0.831, which confirmed a high reliability in applying CCS (17). The tools were rated using Likert's 5-level points, with higher scores indicating better health behavior and more severe constipation.

### Data collection

The distribution and collection of questionnaires were the responsibility of five designated researchers. In order to reduce the bias in the research process, the researchers were able to unify the guidance language and accurately master the knowledge related to functional constipation, scale content and data collection methods. General information about patients can also be obtained through medical care records and archives of the community. Besides, all subjects included in the study completed the 3-month trial, and all data collection was completed.

### Statistical analysis

The statistical analysis of the data obtained from the study was performed using GraphPad Prism(Version 9) software. The measurement data with normal distribution were expressed as mean (M) and standard deviation (SD) and a Student's *t*-test was performed. The enumeration data were statistically described by frequency and percentage and analyzed using the Chi-square test, and *P* < 0.05 was defined as statistically significant.

## Results

### Sociodemographic characteristics and medical status

The Table 2 shows that the majority of participants were female, who accounted for 80.00% of the control group and 83.33% of the experimental group. The average age of participants was 67.46 ± 10.64 years in the control group and 68.59 ± 12.11 years in the experimental group. The BMI calculated by height and weight of the two groups was close to 22. More than 80% of the participants defecated hard and massive stool and could not defecate without laxatives. More than 50% of the participants complained about difficult defecation and abdominal distention. More than 23% of participants had taken calcium antagonists and nitrates for a long time. All the differences were not statistically significant in sociodemographic characteristics and medical data (*P* > 0.05) (Table 2).

**Table 2:** Comparison of general data between the two groups.

**Items**	**Control group (*n* = 30) *n* (%)**	**Experimental group (*n* = 30) *n* (%)**	***P-value***
**Gender**			
Female	24 (80.00)	25 (83.33)	0.48
Male	6 (20.00)	5 (16.67)	
Age, mean SD	62.46 (10.64)	63.59 (12.11)	0.85
BMI, mean SD	23.03 (7.52)	21.40 (6.20)	0.65
Difficult defecation	18 (60.00)	20 (66.67)	0.99
The stool is hard or massive, once every four times	24 (80.00)	26 (86.67)	0.89
Defecate endlessly, once every four times	24 (80.00)	26 (86.67)	0.89
Constipation with abdominal distention	20 (66.67)	25 (83.33)	0.12
Manual assistance, once every four times	14 (46.67)	13 (43.33)	0.79
Can't defecate without laxatives	24 (80.00)	25 (83.33)	0.48
Long term use of calcium antagonists	9 (30.00)	7 (23.33)	0.54
Long term use of nitrates	8 (26.67)	7 (23.33)	0.76

SD, Standard Deviation; BMI, Body Mass Index.

### Health dietary behavior improved after the intervention in HLPCQ

Table 3 presents the comparison of healthy behavior of patients with functional constipation in the experimental and control groups. As shown in the Table 3, the scores before and after the intervention for the same patient were compared. Besides, intra-group comparisons were performed, that is, between control and experimental groups at baseline and between control and experimental groups after intervention. No difference was observed in the baseline health behavior between the two groups (*P* > 0.05). The overall average in the experimental group (77.90 ± 14.57) was significantly greater than that in the control group (61.11 ± 13.64) after the intervention (*P* < 0.05). The scores in four out of five dimensions, including healthy dietary choices (21.48 ± 3.15 vs. 12.55 ± 2.71), dietary harm avoidance (12.68 ± 2.94 vs. 8.53 ± 1.50), daily routine (23.37 ± 4.72 vs. 14.11 ± 3.89) and social and mental balance (14.72 ± 2.27 vs. 11.01 ± 1.64), showed that the empowerment results of the experimental group were significantly better than those of the control group (*P* < 0.05). Among these, 14/26 questions received higher scores in the experimental group than in the control group (*P* < 0.05).

**Table 3:** Comparison of health behaviors before and after the interventions in HLPCQ.

**Item, mean ±SD**	**Control group**			**Experimental group**			**Intra-group** ***P-value***	
	**Baseline**	**After intervention**	***P-value***	**Baseline**	**After intervention**	***P-value***	**Baseline**	**After intervention**
**Overall average**	46.18 ± 6.39	61.11 ± 13.64	<0.01	45.24 ± 5.72	77.90 ± 14.57	<0.01	0.28	<0.01
**Healthy dietary choices**	11.54 ± 2.83	12.55 ± 2.71	0.06	11.51 ± 2.42	21.48 ± 3.15	<0.01	0.31	<0.01
Are you careful about how much food you put on your plate	1.85 ± 0.76	2.14 ± 0.53	0.11	2.00 ± 0.96	4.51 ± 0.63	<0.01	0.26	<0.01
Do you check the food labels before buying a product	1.77 ± 0.64	2.19 ± 0.84	0.03	1.51 ± 0.57	2.15 ± 1.09	0.02	0.79	0.09
Do you calculate the calories of your meals	1.25 ± 0.52	1.48 ± 0.64	0.21	1.27 ± 0.52	4.79 ± 0.49	<0.01	0.34	<0.01
Do you limit fat in your meals	1.96 ± 1.19	2.78 ± 0.80	<0.01	2.00 ± 1.00	2.80 ± 0.97	<0.01	0.37	0.15
Do you like cooking	1.95 ± 0.98	2.15 ± 0.52	0.34	1.90 ± 0.97	2.23 ± 0.95	0.15	0.46	0.48
Do you eat organic foods	1.27 ± 0.46	1.52 ± 0.47	0.08	1.24 ± 0.51	4.72 ± 0.52	<0.01	0.53	<0.01
Do you eat whole-wheat products	1.49 ± 0.79	1.81 ± 0.81	0.06	1.37 ± 0.72	4.68 ± 0.54	<0.01	0.77	<0.01
**Dietary harm avoidance**	7.98 ± 1.48	8.53 ± 1.50	0.07	7.38 ± 1.85	12.68 ± 2.94	<0.01	0.15	0.01
Do you avoid eating packaged- or fast-food	1.51 ± 0.70	2.01 ± 0.72	0.11	1.58 ± 0.68	4.33 ± 0.11	<0.01	0.11	<0.01
Do you avoid soft drinks	1.66 ± 0.73	2.01 ± 0.67	0.06	1.65 ± 0.72	3.21 ± 0.14	<0.01	0.16	0.04
Do you avoid eating when stressed or disappointed	2.59 ± 0.97	4.03 ± 0.37	<0.01	1.96 ± 1.01	3.98 ± 0.59	<0.01	0.37	0.28
Do you avoid binge eating when you are out with friends	2.07 ± 0.95	2.22 ± 0.45	0.43	2.17 ± 1.13	3.01 ± 0.13	<0.01	0.12	0.07
**Daily routine**	13.11 ± 2.77	14.11 ± 3.89	0.12	12.34 ± 2.64	23.37 ± 4.72	<0.01	0.26	<0.01
Do you eat your meals at same time each day	1.55 ± 0.69	2.04 ± 0.17	0.43	1.51 ± 0.63	4.59 ± 0.73	<0.01	0.39	<0.01
Are you careful about not missing a meal	1.70 ± 0.66	1.97 ± 0.58	0.13	1.37 ± 0.56	3.86 ± 0.29	<0.01	0.17	<0.01
Do you eat a good breakfast	1.48 ± 0.57	1.53 ± 0.91	0.77	1.31 ± 0.60	4.99 ± 0.77	<0.01	0.69	<0.01
Do you sleep at the same time each day	2.11 ± 0.93	3.99 ± 0.90	<0.01	1.79 ± 0.86	4.10 ± 0.13	<0.01	0.46	0.68
Do you follow a scheduled program for your daily activities	2.03 ± 0.85	3.96 ± 0.22	<0.01	1.86 ± 0.74	3.95 ± 0.87	<0.01	0.37	0.79
Do you eat breakfast at the same time each day	1.40 ± 0.50	1.49 ± 0.47	0.61	1.58 ± 0.68	4.24 ± 0.13	<0.01	0.78	<0.01
Do you eat lunch at the same time each day	1.99 ± 0.45	2.10 ± 0.35	0.37	1.41 ± 0.68	4.18 ± 0.22	<0.01	0.61	<0.01
Do you eat dinner at the same time each day	1.57 ± 0.57	1.68 ± 0.51	0.37	1.48 ± 1.05	4.36 ± 0.24	<0.01	0.19	<0.01
**Organized physical exercise**	4.04 ± 0.85	5.07 ± 1.26	<0.01	4.07 ± 0.88	5.62 ± 1.59	<0.01	0.45	0.16
Do you practice aerobic exercise for 20 or more minutes at least 3 times per week	2.00 ± 0.78	2.97 ± 0.49	<0.01	1.89 ± 0.90	2.89 ± 0.99	<0.01	0.48	0.69
Do you exercise in an organized manner	2.03 ± 0.75	2.89 ± 0.65	<0.01	2.17 ± 0.80	3.18 ± 0.69	<0.01	0.37	0.52
**Social and mental balance**	10.39 ± 1.68	11.01 ± 1.64	0.07	9.93 ± 1.16	14.72 ± 2.27	<0.01	0.15	<0.01
Do you share your personal problems or worries with others	1.55 ± 0.57	1.87 ± 0.48	0.32	1.37 ± 0.49	4.33 ± 0.16	<0.01	0.28	<0.01
Do you concentrate on positive thoughts during difficulties	1.81 ± 0.73	2.27 ± 0.46	0.01	1.96 ± 0.77	3.39 ± 0.95	<0.01	0.10	0.69
Do you empty your brain of thoughts or the next day's program during bedtime	2.25 ± 0.94	3.19 ± 0.64	<0.01	1.96 ± 0.90	3.18 ± 0.57	<0.01	0.38	0.79
Do you care about meeting and discussing with your family on a daily basis	1.37 ± 0.56	1.59 ± 0.79	0.26	1.37 ± 0.62	4.21 ± 0.53	<0.01	0.46	<0.01
Do you balance your time between work, personal life and leisure	3.33 ± 1.03	3.55 ± 0.86	0.31	3.24 ± 0.98	3.29 ± 1.08	0.82	0.13	0.27

SD, Standard Deviation.

### Symptoms of constipation improved after the intervention in CCS

Table 4 shows that no difference was observed in the baseline constipation symptoms between the two groups (*P* > 0.05). After intervention, the overall mean score of the experimental group decreased to 7.48 ± 3.73, which was less than that of the control group (9.70 ± 3.07) (*P* > 0.05). Significantly, 5/8 clinical problems, including defecation frequency (0.20 ± 0.41 vs. 1.61 ± 0.62), difficult defecation (1.10 ± 1.08 vs. 1.99 ± 1.08), incomplete evacuation (1.17 ± 1.03 vs. 1.99 ± 1.15), straining period of defecation (0.86 ± 0.74 vs. 2.33 ± 1.10) and defecation failure (0.58 ± 0.73 vs. 0.96 ± 0.58), in the experimental group received lower scores than those in the control group (*P* < 0.05).

**Table 4:** Comparison of constipation symptoms before and after the interventions in CCS.

**Item, mean ±SD**	**Control group**			**Experimental group**			**Intra-group** ***P-value***	
	**Baseline**	**After intervention**	***P-value***	**Baseline**	**After intervention**	***P-value***	**Baseline**	**After intervention**
Overall average	15.85 ± 2.94	9.70 ± 3.07	<0.01	16.79 ± 3.17	7.48 ± 3.73	<0.01	2.43	<0.01
Defecation frequency	2.07 ± 1.26	1.61 ± 0.62	0.08	2.27 ± 1.22	0.20 ± 0.41	<0.01	0.27	<0.001
Difficult defecation	2.51 ± 1.07	1.99 ± 1.08	0.05	2.72 ± 0.92	1.10 ± 1.08	<0.01	0.36	0.04
Incomplete evacuation	2.55 ± 1.18	1.99 ± 1.15	0.06	2.48 ± 1.02	1.17 ± 1.03	<0.01	0.4	<0.01
Abdominal pain	1.44 ± 1.01	0.77 ± 0.89	0.02	1.44 ± 1.05	0.86 ± 0.91	0.03	0.49	0.36
Straining period of defecation	2.85 ± 1.32	2.33 ± 1.10	0.08	2.86 ± 1.21	0.86 ± 0.74	<0.01	0.49	0.03
Defecation assistance	1.18 ± 0.55	0.33 ± 0.48	<0.01	1.10 ± 0.61	0.20 ± 0.49	<0.01	0.3	0.16
Defecation failure: Unsuccessful defecation in 24 h	1.22 ± 0.80	0.96 ± 0.58	0.10	1.20 ± 0.90	0.58 ± 0.73	<0.01	0.47	0.01
History of constipation	2.77 ± 0.93	1.92 ± 0.91	<0.01	2.75 ± 0.98	1.48 ± 1.02	<0.01	0.47	0.28

SD, Standard Deviation.

## Discussion

In this study, the majority of patients with functional constipation were women, which is consistent with the domestic and international epidemiological data (18). The reason why women are more likely to suffer from functional constipation than men is mainly related to hormones; that is, it may be caused by the decrease in the colonic smooth muscle motility resulting from the increased level of progesterone (19). The average age of the patients was ~68 years old. Studies have shown that as the colonic epithelium and pelvic floor muscle and nerve cells degenerate with age, the intestinal muscle strength and the secretion of intestinal cells decrease (20); thus, the elderly are more prone to constipation. In addition, some participants used calcium antagonists and nitrates, which is consistent with the literature discussing the positive relevance of constipation and cardiovascular disease (21). In addition, the baseline sociodemographic characteristics, healthy behavior and constipation symptoms of the patients with functional constipation showed no difference between the experimental and control groups.

The overall average scores of healthy behavior of 60 patients with functional constipation were lower than Darviri's survey results on the healthy population (16), indicating the poor overall level of lifestyle behavior and personal control of patients with functional constipation in this study and the need to strengthen their healthy behavior, especially in healthy diet choice, avoid diet injury and daily living habits. Healthy diet choice, avoidance of diet injury and daily life management were mainly reflected in the patients' diet management behavior, indicating that a good diet structure has not been formed. A high dietary fiber diet, as a functional food, has been recommended for the treatment and alleviation of constipation by normalizing the structure of intestinal flora to improve the intestinal ecology (22). Therefore, high dietary fiber food models and cards should be used in education demonstrations to promote the empowering effect for patients with functional constipation.

On the basis of the 24-h review, the patient adhered to our recommendations. After the intervention of empowerment-based behavioral intervention, improvements were observed in the patients' healthy dietary behaviors, such as being careful about the amount of food put on their plate, calculating meal calories, consuming organic foods and whole-wheat products and avoiding packaged or fast food. Individuals who do not eat whole grains, fresh fruits and vegetables every day have a high prevalence of constipation (23). In the dimension of daily routine, the patients' meals were usually irregular; they either eat on time, do not eat, or only eat perfunctorily. This irregular behavior is usually related to the patients' lack of awareness and attention to constipation. Moreover, the modern fast-paced lifestyle exacerbates this behavior (24). In addition, the survey on the social and psychological balance dimensions showed that patients with functional constipation rarely share personal problems or concerns with others nor discuss constipation with their families. Some investigations have shown that psychological stress and social disorder easily cause constipation (25, 26) and anxiety tends to have a negative impact on their daily life (27). However, patients in the control group diagnosed with functional constipation were managed by their responsible nurse and doctor, their specific treatment measures were dominated by medical personnel. Through patient empowerment, patients in the experimental group have exhibited stronger desire for knowledge and interpersonal interaction. Their subjective initiative and willingness to communicate were boosted. The results of this study suggest that only when patients are in a positive psychological state will they take on healthy behavior.

The main purpose of patient empowerment is to encourage patients to adopt healthy dietary behavior and the final outcome is the improved symptoms of constipation. Compared with the control group, the constipation symptoms of patients in the experimental group, including defecation frequency, difficult defecation, incomplete evacuation, straining period of defecation and defecation failure, significantly improved. Rasmussen reported that the defecation frequency during daytime is the most prominently associated with health-related quality of life (28). Therefore, empowering patients' health dietary behavior, from a long-term perspective, is conducive to improving patients' constipation symptoms and quality of life. Nevertheless, dealing with a larger scale and different types of patients with functional constipation, how to improve the healthy dietary behavior of patients through high-efficiency empowerment intervention deserves further research. Overall, the results of this study have shown that patient empowerment can strengthen subjective initiative and healthy behavior of elderly patients with functional constipation and promote active participation in self-care. Future research is also recommended to test the effectiveness of the empowerment-based intervention with an educated demonstration among a broad range of population groups.

## Conclusion

This study showed that empowerment-based intervention plays a considerable role in improving the healthy dietary behavior of elderly patients with functional constipation. The intervention yielded a significant positive performance in the experimental group, addressed the problems experienced by elderly patients due to functional constipation, resulted in a decreased constipation severity and improved the quality of life. Besides, strengthening the subjective initiative of elderly patients through patient empowerment can reduce patients' over-dependence on medical resources. Therefore, this study provides a non-pharmacological treatment reference for elderly patients suffering from functional constipation.

## Data availability statement

The original contributions presented in the study are included in the article/supplementary material, further inquiries can be directed to the corresponding author.

## Ethics statement

The studies involving human participants were reviewed and approved by the Ethics Committee of Wuxi Third People's Hospital (Renamed later as Affiliated Hospital of Jiangnan University) (No. IEC201803001). The patients/participants provided their written informed consent to participate in this study.

## Author contributions

XZ, FZ, DL, and JC designed the trial. YX and XZ conducted the study. YX, XZ, DL, and QW analyzed and interpreted the data. XW, FZ, DL, and HC were responsible for project administration and supervision. XW, XZ, FZ, and DL wrote the first draft of the manuscript and had primary responsibility for the manuscript's final content. All authors critically revised the manuscript. All authors contributed to the article and approved the submitted version.
